# Is manipulative therapy more effective than sham manipulation in adults?: a systematic review and meta-analysis

**DOI:** 10.1186/2045-709X-21-34

**Published:** 2013-10-02

**Authors:** Gwendolijne GM Scholten-Peeters, Erik Thoomes, Sophie Konings, Michelle Beijer, Karin Verkerk, Bart W Koes, Arianne P Verhagen

**Affiliations:** 1 University of Applied Sciences AVANS, Research Group Diagnostics, PO Box 90116, Breda, RA 4800, The Netherlands; 2 Department of General Practice, Erasmus Medical Centre, University Medical Center, PO Box 2040, Rotterdam, CA 3000, The Netherlands; 3 Rotterdam University of Applied Sciences, Rotterdam, Department physical therapy, Museumpark 40, Rotterdam, CX 3015, The Netherlands

**Keywords:** Spinal manipulation, Musculoskeletal manipulation, Manipulative therapy, Systematic review, Sham, Efficacy, Adverse effects

## Abstract

**Background:**

Manipulative therapy is widely used in the treatment of spinal disorders. Manipulative techniques are under debate because of the possibility of adverse events. To date, the efficacy of manipulations compared to sham manipulations is unclear. The purpose of the study is: to assess the efficacy of manipulative therapy compared to sham in adults with a variety of complaints.

**Study design:**

Systematic review and meta-analysis.

**Methods:**

Bibliographic databases (PubMed, EMBASE, CINAHL, PEDro, Central) along with a hand search of selected bibliographies were searched from inception up to April 2012.

Two reviewers independently selected randomized clinical trials (RCTs) that evaluated manipulative therapy compared to sham manipulative therapy in adults, assessed risk of bias and extracted data concerning participants, intervention, kind of sham, outcome measures, duration of follow-up, profession, data on efficacy and adverse events. Pooled (standardized) mean differences or risk differences were calculated were possible using a random effects model. The primary outcomes were pain, disability, and perceived recovery. The overall quality of the body of evidence was evaluated using GRADE.

**Results:**

In total 965 references were screened for eligibility and 19 RCTs (n = 1080) met the selection criteria. Eight studies were considered of low risk of bias. There is moderate level of evidence that manipulative therapy has a significant effect in adults on pain relief immediately after treatment (standardized mean difference [SMD] - 0.68, 95% confidence interval (-1.06 to -0.31). There is low level of evidence that manipulative therapy has a significant effect in adults on pain relief (SMD - 0.37, -0.69 to -0.04) at short- term follow-up. In patients with musculoskeletal disorders, we found moderate level of evidence for pain relief (SMD - 0.73, -1.21 to -0.25) immediate after treatment and low level of evidence for pain relief (SMD - 0.52, -0.87 to -0.17) at short term-follow-up. We found very low level of evidence that manipulative therapy has no statistically significant effect on disability and perceived (asthma) recovery. Sensitivity analyses did not change the main findings. No serious adverse events were reported in the manipulative therapy or sham group.

**Conclusions:**

Manipulative therapy has a clinical relevant effect on pain, but not on disability or perceived (asthma) recovery. Clinicians can refer patients for manipulative therapy to reduce pain.

## Background

Manipulative therapy (MT) is widely used in the treatment of musculoskeletal and other kind of complaints. Its use has increased over the world in the past few decades [1]. Manipulative therapy consists of manipulations, which are passive, high velocity, low amplitude thrusts applied to a joint complex within its anatomical limit (active and passive motion occurs within the range of motion of the joint complex and not beyond the joint’s anatomic limit). The intent of a manipulation is to create motion (including articular surface separation), function, and/or to reduce pain. It is often accompanied by a brief or repetitive popping noise within the affected joint [2]. The cracking sound is caused by cavitation of the joint, which is a term used to describe the formation and activity of bubbles within the fluid [3,4]. The mechanisms through which manipulations may alter musculoskeletal pain are unknown. Current evidence suggests an interaction between mechanical factors such as movement and forces and associated neurophysiological responses to these mechanical factors [5,6]. Various practitioners, including manipulative physical therapists, physicians, chiropractors or osteopaths use these interventions. However, the theoretic hypothesis, diagnostic tools and treatment methods between the professions differ considerably [7].

In the literature there have been reports published about an apparent association between cervical manipulation and serious complications such as arterial dissection and subsequent stroke, while others found no relation [8-13]. Minor adverse events such as aggravation of neck pain or headache, muscle soreness or stiffness are reported more often following manipulation [14]. Ideally to be justified, the risk-benefit ratio of (cervical) manipulations should be known. Manipulative therapy could be used if there is a substantial benefit that exceeds the risks (and costs). To provide insight into the active agent of manipulative therapy, research about the efficacy is needed. These trials will represent an attempt to differentiate between specific and non-specific therapeutic effects of manipulative therapy.

As far we know there are no systematic reviews published about the efficacy of manipulative therapy versus sham manipulative therapy in adults with a variety of complaints. Earlier systematic reviews evaluated manipulative therapy versus other conservative treatments, waiting list controls or sham in specific patient groups such as low back pain, asthma or dysmenorrhea [15-17]. Therefore, the aim of this systematic review was to evaluate the efficacy of manipulative therapy compared with 'sham manipulative therapy’ in adults with a variety of complaints on pain, disability or perceived recovery immediate after treatment, at the short term and long term follow-up.

## Methods

### Selection criteria

We consider published randomised clinical trials (RCTs) studies eligible that stated to evaluate manipulative therapy, including manipulations (as defined by the original authors), compared to sham manipulative therapy in adult participants (18 years of age or older) with a diversity of complaints. Studies were selected that used at least one of our primary outcome measures namely, pain intensity, disability or perceived recovery. Functions (e.g. range of motion, endfeel, propriocepsis, pulmonary functions), adverse events, quality of life and return to work were considered as secondary outcomes.

### Search strategy

We identified RCTs by electronically searching the following databases from inception until April 2012: MEDLINE, EMBASE, CENTRAL (The Cochrane Library April 2012), CINAHL and PEDro. The sensitive search strategy developed by the Cochrane Handbook for Systematic Review of Interventions was followed, using free text words and MeSH Headings (Medline), Thesaurus (EMBASE, CINAHL) [18]. Combinations were made based on a) intervention (manipulation, spinal manipulation, manipulative therapy, high velocity thrust, chiropractic manipulation, osteopathic manipulation, musculoskeletal manipulation), b) comparison (placebo, sham treatment, sham manipulation and c) design: randomised clinical trial or randomised controlled trial. The complete search strategy is available on request from the primary review author. References from the included studies as well as relevant systematic reviews were screened and experts approached in order to identify additional studies. One research librarian together with a review author (WS) performed the electronic searches. Two review authors (WSP, ET) independently selected the studies first by screening title and abstract, and secondly by screening the full text papers. No restrictions were applied to year of publication or language. Disagreements on inclusion were resolved by discussion or through arbitration by a third review author (AV).

### Risk of bias assessment

Two review authors (WSP, ET) independently assessed the risk of bias (RoB) of the included RCTs using the 12 criteria recommended by the Cochrane Back Review Group [18]. The criteria were scored as “yes,” “no,” or “unclear” and reported in the *Risk of Bias* table. Disagreements were solved in a consensus meeting. When disagreement persisted, a third review author (AV or KV) was consulted. A study with a low RoB was defined as fulfilling six or more of the criteria items, which is supported by empirical evidence [19].

### Data extraction

Two review authors (WSP, ET, SK and MB) independently extracted the data using a standardized form (including profession, participants, intervention, kind of sham, outcome measures, duration of follow-up, drop-outs, data on efficacy and adverse events). Follow-up time intervals were defined as immediate (within one day), short-term (≤ 3 months) and long-term (≥ 6 months). In cases of uncertainly about the data extracted, a third review author (AV) was consulted.

### Data analysis

The inter-observer reliability of the risk of bias assessments was calculated using Kappa statistics and percentage agreement. We assessed the possibility of publication bias by creating funnel plots. For continuous data, we calculated weighted mean differences (WMD) with 95% confidence intervals (95% CI). Visual Analogue Scales (VAS) or Numerical Pain Rating Scales (NPRS) were converted to a 100-point scale, when necessary. In case different instruments were used to measure the same clinical outcome, we calculated standardized mean differences (SMD). For dichotomous outcomes, we calculated Risks Differences (RD) and 95% CI. All analyses were conducted in Review Manager 5.1, using a random-effects model. Prior to pooling, clinical heterogeneity sources were assessed such as participants, time-frame and outcomes. Statistical heterogeneity was considered using a cut-off point of 50%; then the results were thought to be too heterogeneous to pool. Stratified analyses were considered: 1) by time (immediate, short-term, long- term); 2) type of participants (musculoskeletal complaints versus non-musculoskeletal complaints); 3) profession (chiropractor, physical therapist, osteopath, physician). We planned sensitivity analyses a priori to explain possible sources of heterogeneity for RoB. Results are considered clinically relevant when the pooled SMD is at least ≥ 0.5 [20].

### Strength of the evidence

The overall quality of the evidence and strength of recommendations were evaluated using GRADE (Grading of Recommendations Assessment, Development and Evaluation) [21]. The quality of the evidence was based on performance against five principal domains: (1) limitations in design (downgraded when more than 25% of the participants were from studies with a high RoB), (2) inconsistency of results (downgraded in the presence of significant statistical heterogeneity [*I*^2^ > 50%] or inconsistent findings (defined as ≤75% of the participants reporting findings in the same direction), (3) indirectness (e.g. generalizability of the findings; downgraded in those studies that used a specific subset of the population under investigation), (4) imprecision (downgraded when the total number of participants was less than 400 for continuous outcomes and 300 for dichotomous outcomes), and (5) other considerations, such as publication bias [21].

High quality evidence was defined as RCTs with low risk of bias that provided consistent, direct and precise results for the outcome. The quality of the evidence was downgraded when one of the factors described above was met [21]. Two independent review authors (WSP, ET) graded the quality of evidence. Single studies (N < 400 for continuous outcomes, N < 300 for dichotomous outcomes) were considered inconsistent and imprecise (i.e. sparse data) and provide “low quality evidence”, which could be further downgraded to “very low quality evidence” if there were also limitations in design or indirectness [21]. The following grading of quality of the evidence was applied:

•High quality: further research is very unlikely to change our confidence in the estimate of efficacy;

•Moderate quality: further research is likely to have an important impact on our confidence in the estimate of efficacy and may change the estimate; one of the domains is not met;

•Low quality: further research is very likely to have an important impact on our confidence in the estimate of efficacy and is likely to change the estimate; two of the domains are not met;

•Very low quality: we are very uncertain about the estimate; three of the domains are not met.

## Results

### Results of the search

**Figure 1:**
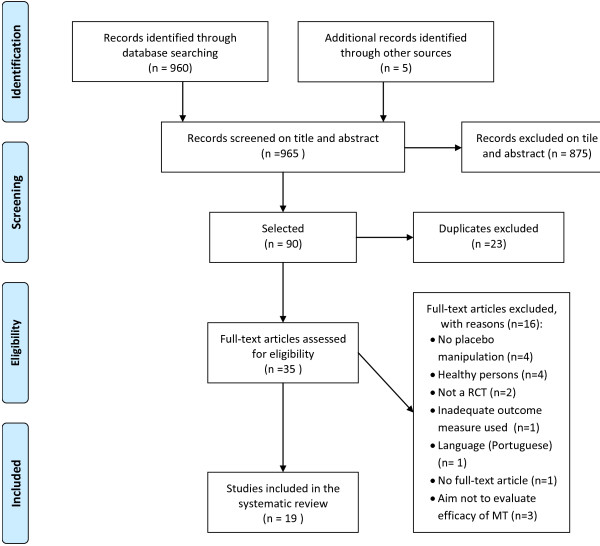
Flowchart of the inclusion and exclusion of RCTs.

A total of 965 titles and abstracts were screened, of which 35 full text articles were selected (Figure 1). After screening the full text articles and searching bibliographies of included studies and systematic reviews, 19 papers were identified and included [22-40], and one study could not be assessed because of the language (Portuguese) [41]. Any differences between the two review authors were resolved by consensus.

### Description of studies

Table 1 represents the characteristics of the 19 studies included in the review.

**Table 1:** Study characteristics

**Author (year) profession**	**Patient population**	**Intervention (n) follow-up**	**Sham specification**	**Outcome (instrument)**	**Results mean, sd (n) and WMD with 95% CI or n and RD with 95 CI**	**Author’s conclusion**
**Brantingham et al (2003)**[22]	Osteoarthritis of the hip (n = 8)	I1: hip manipulations (4)	Sham manipulation with deactivated Activator Instrument a spring loaded piston activated instrument to posterior superior iliac spine, iliac crest and greater trochanter	Pain (NRS)	*Final treatment*	MT may have noteworthy short term clinical benefit over sham.
		I2: sham manipulation of the hip (4)		Disability (WOMAC)	Pain: I1: 18.8 (4); I2: 48.8 (4)	
Chiropractic	Mean age:			Hip function (ROM)	Disability: I1: 7.3 (4); I2 37.5 (4)	
	I1: 60 ± 4; I2: 57 ±12	Six treatments over 3 weeks and a one month follow-up visit			ROM flexion: I1: 109.5 (4); I2: 94.3 (4)	
		Follow-up 7 weeks			ROM extension: I1: 10.0 (4); I2: 6.3 (4)	
					*7 weeks follow-up*	
					Pain: I1: 15.0 (4); I2: 36.6 (4)	
					Disability: I1: 7.4 (4); I2: 31.4 (4)	
**Cleland et al (2005)**[23]	Mechanical neck pain (n = 36)	I1: thoracic spine manipulation (19)	Participants in the exact same position as the MT group, deep inhalation and then exhale; no high-velocity low amplitude thrust .	Pain (VAS)	*Immediate*	A statistical significant improvement in pain in the MT group.
		I2: sham manipulation (17)			Pain: I1: 26.1 ± 17.2 (19); I2: 43.5 ± 19.5 (17); WMD: -17.4 (-29.8 to -5)	
Physical therapy	Mean age:					
	I1: 36 ± 8.5;	One intervention				
	I2: 35 ± 11.3	5 minutes post treatment				
**Ghroubi et al (2007)**[24]	Chronic low back pain (n = 64)	I1: spinal manipulation (32)	Sham manipulations under the same conditions as for I1 without the manipulative thrust	Pain (VAS)	*1 month follow-up*	Patients receiving the manipulations showed improvement in pain and disability.
		Four interventions		Disability	Pain: I1: 49.4 ± 16.8 (32); I2: 58.4 ± 28.8 (32); WMD: -9 (-20.8 to 2.8)	
Physical therapy	Mean age:	I2: sham manipulation (32)		(Oswestry)	*2 months follow-up*	
	I1: 39.1 ±11.1; I2: 37.4 ±7.5	One intervention			Pain: I1: 48.1 ± 22.8 (32); I2: 54.4 ± 25.8 (32); WMD: -6.3 (-18.5 to 5.9)	
		Follow-up at 1 and 2 months			Disability: I1: 12.3 ± 5.7 (32); I2: 12.1 ± 5.6 (32); WMD: 0.2 (-2.6 to 3)	
**Hawk et al (2002)**[25]	Chronic Pelvic Pain, (n = 39)	I1: lumbar spine flexion-distraction and trigger point therapy (20)	Sham manipulation with a hand-held adjusting instrument combined with light effleurage	Pain (VAS) Disability (PDI) Quality of life (SF-36)	Median change scores [range] (n)	Mean change scores were not consistent across sites so results were not combined and overall effect sizes were not estimated
**Chiropractic**	Mean age:	I2: sham manipulation and effleurage (19)			*6 weeks follow-up*:	
	I1: 34.7 ± 7.6; I2: 33.7 ± 7.6	Six weeks of treatment with three visits a week for 2 weeks and twice a week for 4 weeks (total14 treatments at 3 different sites/practices)			Pain: I1 site 1: 36 [0, 76] (9); I1 site 2: 20 [-16,50] (6); I1 site 3: -63 [-70, -3.0] (3); I2: site 1: 14 [-12,34] (7); I2 site 2: 11 [-5,60] (6); I2 site 3: 13 [-17,28] (5)	
		Follow-up 12 weeks			Disability: I1 site 1: 9 [1,20] (9); I1 site 2: 7 [-5,21] (6); I1 site 3: 1 [-30,22] (3)	
					I2: site 1: 4 [-18,32] (7); I2 site 2: 8 [2,15] (6); I2 site 3: 14 [1,28] (5)	
					*12 weeks follow-up*:	
					Pain: I1 site 1: 36 [-6, 76] (9); I1 site 2: 23 [-40,51] (6); I1 site 3: -23 [-49,2] (3)	
					I2: site 1: 11 [-29,35] (7); I2 site 2: 4 (-14,38) (6); I2 site 3: 3 (-35,39) (5)	
					Disability: I1 site 1: 9 [0,27] (9); I1 site 2: 9.5 [-14,16] (6); I1 site 3: 2 [-39,25] (3); I2: site 1: 7 [-10,46] (7); I2 site 2: 10.5 [0,19] (6); I2 site 3: 5 [1,20] (5)	
**Hawk et al (2005)**[26]	Subacute (4-12 weeks) or chronic low back pain (more than 12 weeks), (n = 111)	I1: lumbar spine flexion-distraction and trigger point therapy (54)	Sham manipulation with a hand-held adjusting instrument combined with light effleurage	Improvement of symptoms	Improvement symptoms	Patients in both groups improved on RMQ but there were no significant differences
		I2: sham manipulation and effleurage (57)		Disability (RMQ)	I1: n = 29; I2: n = 20 RD: 0.19 (0.0 to 0.37)	
Chiropractic	Mean age:	Eight treatment over 3 weeks		Quality of life (SF-36)	*3 weeks follow-up*	
	I1: 51 ± 14.2; I2: 53 ±15.2	Follow-up 3 weeks			Disability:	
					I1: 1.6 ±3.2 (n = 54); I2: 2.1 ± 3.3 (n = 52)	
					WMD: -0.5 (-1.8 to 0.8)	
**Hondras et al (1999) **[27]	Primary dysmenorrhea, (n = 138)	I1: spinal manipulation (69)	The low force mimic maneuver consisted of positioning the subject on one side with bilateral flexion of the hip and knee joints.	Pain (VAS)	*Mean pre- post change cycle 2*	There are no statistically significant differences between the two interventions.
Chiropractic		I2: low force mimic (69)			Pain: I1: 10.1 ± 14.8 (68); I2: 8.0 ± 16.6 (69)	
	Mean age:	Treatment took place on day 1of cycles 2, 3 and 4.			WMD: 2.1 (-3.2 to 7.4)	
	I1: 31.1; I1: 29.7	Follow-up after each of 4 menstrual cycles.				
**Kokjohn et al (1992)**[28]	Primary dysmenorrhea, (n = 45)	I1: spinal manipulation (24)	Positioning the subject on one side with bilateral flexion of the knee and hip joint; to minimize the mechanical effect	Pain (VAS)	Mean differences	MT is effective in relieving pain
		I2: sham manipulation (21)			Abdominal pain: I1: 20.91 ± 23.0 (23); I2: 8.1 ±15.0 (21); WMD: 12.8 (0.9 to 24.7)	
	Mean age: 30.3	One intervention, Post-treatment			Back pain: I1: 18.7 ±19.4 (23); I2: 7.8 ± 15.7 (21); WMD: 10.9 (0.09 to 21.7)	
**Learman et al (2009)**[29]	Chronic low back pain, (n = 33)	I1: first spinal manipulation second sham procedure(17)	Sham procedure was done in de manipulative position to simulate a manual technique	Pain (VAS) Trunk proprioception (Biodex system 3)	No data	MT had minimal immediate effect on trunk proprioception.
Physical therapy	Mean age:	Two interventions				
	I1: 37.4 ±9.21; I2: 37.25 ±8.65	I2: first sham procedure second spinal manipulation third sham (16)				
Crossover design		Three interventions				
		Intervention took place in a period of one week.				
		Post treatment and 1 week follow-up				
**Licciardone et al (2003)**[30]	Chronic low back pain, (n = 91)	I1: osteopathic manipulative treatment (48)	Subject receiving manipulation as a simulated osteopathic manipulative techniques	Pain (VAS) Disability (RMQ)	No data	Both groups scored better than the control group. No significant benefits were measured.
Osteopathic	Mean age:	Seven interventions				
	I1: 49 ±12; I2: 52 ±12; I3: 49 ±12	I2: sham manipulation (23)				
		Seven interventions				
		I3: no intervention (20)				
		Follow-up at 1, 3 months				
**Mansilla-Ferragut et al (2009)**[31]	Mechanical neck pain, (n = 37)	I1: spinal manipulation of the atlanto-occipital joint (18)	Manual contact intervention similar to cervical thrust manipulation. Head was rotated and maintained for 30 sec.	Pressure pain threshold (Mechanical pressure algometer, kg/cm^2^)	Pressure pain threshold	MT group scored better on pressure pain and active mouth opening
	Mean age:	One intervention		Function: (Active mouth opening in mm)	I1: 0.9 ± 0.3 (18)	
Physical therapy	I1: 36 ±7; I2: 34 ± 8	I2: manual contact sham intervention (19)			I2: 0.7 ± 0.4 (19)	
		One intervention			WMD: 0.2 (-0.04 to 0.4)	
					Function	
					I1: 38.8 ± 4.5 (18)	
					I2: 35.9 ± 4.3 (19)	
					MD: 2.9 (-0.1 to 5.9)	
		5 minutes post-treatment				
**Nielsen et al (1995)**[32]	Chronic asthma, (n = 31)	I1: Active chiropractic spinal manipulation followed by sham (16)	A drop table was used. Gentle pressure over the spinal contact point with one hand was applied, while the other hand trusted on the drop section with the purpose of releasing it.	Perceived recovery (VAS) Pulmonary functions (FEV1, FCV)	Mean change from baseline	No significant differences between MT and sham in perceived recovery and lung functions.
Chiropractic	Mean age: 28.6 ±7.2	I2: Sham chiropractic spinal manipulation followed by active spinal manipulation (15)			Recovery: I1: - 5.93 (16); I2: - 8.46 (15)	
Crossover study		Started with twice a week for a 4-week period. Two weeks cross over.			*FEV*_*1*_*:* I1: 0.05 (16); I2: 0.09 (15)	
					FVC: I1: 0.13 (16); I2: 0.12 (15)	
**Noll et al (2008)**[33]	Elderly patients with obstructive pulmonary disease, (n = 35)	I1: seven osteopathic manipulative techniques 'commonly used for respiratory disorders’ (18)	Light touch at the same anatomic regions in the same position as the manipulative group	Pulmonary functions (FEV1, FVC, RAW, residual volume)	*Post-treatment*	Overall worsing of air trapping immediate after manipulation compared to sham.
Osteopathic		I2: sham manipulative techniques (17)			FEV1: I1: 1.18 ±0.62 (18); I2: 1.28 ± 0.63 (17); WMD: -0.1 (-0.5 to 0.3)	
	Mean age:				FVC; I1: 2.36 ± 0.93 (18); I2: 2.66 ± 0.92 (17); WMD: -0.3 (-0.94 to 0.34)	
	I1: 69.6 ± 6.6; I2: 72.2 ± 7.1	One intervention.			FEF25-75 L/sec; I1: 0.43 ± 0.31 (18)	
		Post treatment and 1 day follow-up with a survey			I2: 0.55 ± 0.43 (17); WMD: 0.12 (-0.14 to 0.38)	
					RAW: I1: 6.15 ± 5.22 (18); I2: 7.71 ± 6.09 (17); WMD: -1.6 (-5.5 to 2.3)	
					Residual volume; I1: 5.02 ± 3.06 (18)	
					I2: 4.84 ± 1.84 (17); WMD: 0.18 (-1.6 to 2.0)	
**Sanders et al (1990)**[34]	Acute low back pain < 2 weeks, (n = 18)	I1: MT L4/L5-S1 region (6)	Light physical contact/touch at the L4/L5-S1 region of the spine	Pain (VAS)	No data	Significant reduction of pain in de manipulation group, not in the other groups. No between group analyses.
Chiropractic		I2: sham manipulation L4/L5-S1 (6)				
	Mean age:	I3: no treatment or physical contact (6)				
	Males 41 ± 13.9; Female 33 ± 8.6	One intervention. 5 and 30 minutes post treatment				
**Santilli et al (2006)**[35]	Acute low back pain and sciatica with disc protrusion on resonance imaging, (n = 102)	I1: soft tissue manipulations and rotational MT (53)	Soft muscle pressing similar to MT but not following any specific patterns and not involving rapid thrusts	Pain (number of patients pain-free) at end of follow-up Quality of life (SF-36)	*180 days follow-up*	Active manipulations are more effective than sham on percentage pain-free cases, not on SF-36 scores
Chiropractic		I2: soft muscle pressing (49)			Pain: low back pain; I1: n = 15; I2: n = 3	
	Mean age: 43.1	Maximum of 20 sessions, 5 days per week			RD: 0.22 (0.08 to 0.36)	
		Follow-up 15-30-45-90-180 days after first visit			Referred pain; I1: n = 29 (48*)*; I2: n = 10 (48) RD: 0.34 (0.17 to 0.52)	
					Quality of life; I1: 53.8 ±16.8 (53)	
					I2: 57.5 ± 20 (49)	
					WMD: -3.7 (-10.9 to 3.5)	
**Senna and Machaly (2011)**[36]	Chronic nonspecific low back pain, (n = 93)	I1: maintained MT and ROM exercise (26)	Manually applied force of diminished magnitude, aimed purposely to avoid treatable areas of the spine.	Pain (VAS) Disability (Oswestry)	*1 month follow-up*	After 1 and 10 months the subjects receiving maintenance MT had lower pain and disability scores and higher quality of life scores compared to sham.
	Mean age:	I2: sham manipulation and ROM exercise (40)		Quality of life (SF-36)	Pain: I1: 29.4 ± 5.5 (25); I2: 33.2 ± 7.3 (37); I3: 29.5 ± 6.1 (26)	
Physician	I1: 41.6 ±11; I2: 42.4 ±9.7; I3: 40.3 ± 11.7	I3: non-maintained MT and ROM exercise (27)			WMD (I1 vs I2): -3.8 (-7.2 to -0.4)	
		I2 and I3 12 treatments of MT or sham MT over 1 month period in I2 and I3.			Disability: I1: 24.6 ± 8.0 (25); I2: 32.5 ± 12.8 (37); I3: 24.1 ± 9.2 (26)	
		I1 received the same treatments of MT as I3 and additional MT every two weeks for the next 9 months.			WMD (I1 vs I2): -7.9 (-13.7 to – 2.1)	
		Follow-up 1,4,7,10 months			Quality of life: I1: 32.1 ± 7.0 (25); I2: 27.1 ± 7.9 (37); I3: 31.6 ± 8.2 (26)	
					WMD (I1 v I2): 5.0 (1.1 to 8.9)	
					*10 months* Pain: I1: 23.5 ± 8.0 (25); I2: 38.3 ± 12.8 (37); I3: 38.5 ± 12.8 (26)	
					WMD (I1 vs I2): -14.8 (-20.6 to -9.0)	
					Disability: I1: 20.6 ± 7.5 (25); I2: 37.4 ± 13.4 (37); I3: 34.9 ± 12.2 (26)	
					WMD (I1 vs I2): -16.8 (-22.7 to -10.9)	
					Quality of life: I1: 33.7 ± 7.0 (25); I2: 25.9 ± 7.9 (37); I3: 27.7 ± 8.2 (26)	
					WMD (I1 v I2): 7.8 (3.9 to 11.7)	
**Vernon et al (2009)**[37]	Tension type headache, (n = 20)	I1: amitriptyline and MT (4)	A treatment table with a head piece that was capable of a small downward displacement (drop-piece). Drop-piece was quickly engaged simulating the thrust. Before brief preparatory soft tissue massage.	Days of headache reduction in the last 28 days of the trial (headache diary)	I1: -8.4 ± 7.5 (4)	Combined treatment of chiropractic and amitriptyline showed significant and clinical relevant results in headache reduction
		I2: amitriptyline and sham MT (5)			I2: 3.1 ± 5.4 (5)	
Chiropractic	Mean age:	I3: sham amitriptyline and MT (6)			I3: 2.0 ± 6.3 (6)	
	I1: 29 ± 9.8; I2: 29.4 ± 10.1; I3: 34 ± 11.6; I4: 43 ± 4.5	I4: sham amitriptyline and sham MT (5)			WMD (I1 v I2): -11.5 (-21.6 to – 1.4)	
		Chiropractic MTafter 4 weeks of amitriptyline, 3 times/week for 6 weeks and then once per week for 4 weeks.				
		Follow-up 4, 10, 14 weeks				
**Waagen et al (1986)**[38]	Chronic low back pain (> 3 weeks), (n = 29)	I1: MT (11)	Lumbar drop-piece on the chiropractic adjusting table to minimal tension. Adjustment by applying gentle pressure over posterior superior iliac spines.	Pain (VAS) Function: lumbar spine function tests	Pain: *immediate* (mean differences between pre-post)	MT is effective for relieving pain compared to sham MT
		I2: sham MT (18)			I1: 13 (9); I2: 7 (10)	
**Chiropractic**		Two of three times weekly for 2 weeks			*2 weeks*; Pain: I1: 23 (9); I2: 6 (10)	
	Mean age:	Follow-up after 2 weeks			ASLR: I1: 6 ± 8.7 (9); I2: -13.5 ± 10.3 (8); WMD: 19.5 (9.7 to 29.4)	
	I1: 25.2; I2: 24.3				Flexion; I1: 0.34 ± 0.9 (9); I2: 0.95 ± 2.2 (8); MD: -0.6 (-2.3 to 1.1)	
					Extension; I1: 1.2 ± 1.2 (9); I2: -0.5 ± 2.1 (8); WMD: 1.7 (-0.04 to 3.4)	
**Walsh and Polus (1999)**[39]	Premenstrual syndrome (PMS), (n = 45)	I1: first high velocity, low amplitude MT plus soft tissue therapy second sham treatment (28)	The sham treatment used a Activator Adjusting Instrument (Activator Methods Inc., Phoenix, Ariz)	PMS symptoms (PMS-cator disc)	PMS symptoms	For the total group, there was a decrease in the mean global scores in the treatment phase compared with both the baseline and the sham phases
Crossover study	Mean age:	I2: first sham treatment second high velocity, low amplitude MT plus soft tissue therapy (17)			I1: 34.9 ± 25.3 (25)	
Chiropractic	I1: 35 ±7.4; I2: 36 ±7.0				I2: 43.11 ± 26.2 (25)	
		Three times over a period of ten days. Follow-up after 3 menstrual cycles.			WMD: -8.2 (-22.8 to 6.4)	
**Whittingham and Nilsson (2001)**[40]	Cervicogenic headache, (n = 105)	I1: first manipulation, second no treatment, third sham manipulation (56)	Sham manipulation was delivered with a deactivated pettibon instrument	Active cervical ROM (goniometer)	*6 weeks* Right Rotation	Spinal manipulation of the cervical spine increases active range of motion
	Mean age:	I2: first sham manipulation, second manipulation, third no treatment (49)			I1: 67 ± 9.0 (56); I2: 57 ± 9.8 (49)	
Crossover study	I1:39.4 ±11.6; I2:41.9 ±12.5				WMD: 10.0 (6.4 to 13.6)	
Chiropractic		4 study phases in 12 weeks			Left Rotation; I1: 67 ± 9.0 (56); I2: 56 ± 9.8 (49) WMD: 11.0 (7.4 to 14.6)	
		Follow-up 3, 6, 9 and 12 weeks			Right lateral flexion*;* I1: 46 ± 8.2 (56)	
					I2: 39 ± 7.7 (49); WMD: 7.0 (3.9 to 10.1); Left lateral flexion; I1: 44 ± 9.0 (56); I2: 39 ± 9.1 (49); WMD: 5.0 (1.5 to 8.5)	
					*12 weeks* Right Rotation	
					I1: 70 ± 8.0 (53); I2: 73 ± 9.1 (49)	
					WMD: -3 (-6.4 to 0.4)	
					Left Rotation*;* I1: 69 ± 8.0 (53)	
					I2: 72 ± 11.2 (49); WMD: -3 (-6.8 to 0.8); Right Lateral Flexion*;* I1: 47 ± 8.0 (53); I2: 40 ± 9.8 (49); WMD: 7.0 (3.5 to 10.5); Left Lateral Flexion*;* I1: 45 ± 8.0 (53); I2: 47 ± 9.1 (49); WMD: -2.0 (-5.4 to 1.4)	

MT: manipulative therapy, CI: confidence interval, RD: risk difference, ROM: range of motion, VAS-score: visual analogue scale (100 mm), I1: intervention one, I2: intervention two, I3: intervention three, I4: intervention 4, WMD: weighted mean difference, LFM: low force mimic, NRS: numeric rating scale, FEV1: forced expiratory volume 1 second, FVC: forced vial capacity, RAW: airway resistance, ODI: Oswestry Disability Index, PMS: premenstrual syndromes, WOMAC: Western Ontario and McMaster Universities Osteoarthritis Index, SF-36: Short Form Health Survey, RMQ: Roland Morris Back Pain Questionnaire, ASLR: Active Straight Leg Raising test.

#### ***Interventions***

In total, 11 of the 19 studies were identified which compared MT only versus sham [22-24,27-29,31-33,38,40]. Three studies combined MT with soft tissue therapy [39] or trigger point therapy [25,26] and compared this with sham MT and effleurage [25,26]. One study compared range of motion exercises with MT versus range of motion exercises with sham MT [36]. Three studies had three intervention groups [30,34,36], two of them included a no treatment group [30,34]. One study used four intervention groups and a double dummy design evaluating (sham) amitriptyline with (sham) MT [37].

Treatment was delivered by a chiropractor in 12 studies [22,25-28,32,34,35,37-40], a manipulative physical therapist in 4 studies [23,24,29,31], an osteopath in two studies [30,33], and a physician in one study [36].

#### ***Study population***

In total, 1080 patients were included in this systematic review. Sample sizes per study group ranged from 4 to 69 patients. Thirteen studies included less than 25 patients in their smallest study group [22,23,25,28-34,37-39]. Patients were treated either for neck pain [23,31], osteoarthritis [22], chronic pelvic pain [25], chronic low back pain [24,26,29,30,36,38], primary dysmenorrhea [27,28], chronic asthma [32], obstructive pulmonary disease [33], acute low back pain [34], acute low back pain with sciatica [35], tension type headache [37], premenstrual syndrome [39], and cervicogenic headache [40].

#### ***Outcome measures***

Fourteen studies examined pain [22-31,34-36,38], 13 of them used a VAS or NRS [22-30,34-36,38]. Six studies examined disability [22,24-26,30,36], and one study examined perceived (asthma) recovery [32]. Secondary outcomes ranged from quality of life [25,26,35,36], range of motion [22, 38 40], headache frequency [37], to pulmonary function tests [32,33]. Eight studies reported on adverse events [23,27,31-33,35-37]. No studies reported on return-to-work.

#### ***Follow-up measurement***

Most studies examined short-term outcomes [22,24-26,29,30,32,37-40], ranging from 1 week [29] to 3 months [25,30,37,39,40]. Five studies examined the immediate effects of MT [23,28,31,33,34]. Only three studies examined long-term outcomes [27,35,36], ranging from 4 months [27] to 10 months [36].

### Risk of bias

**Figure 2:**
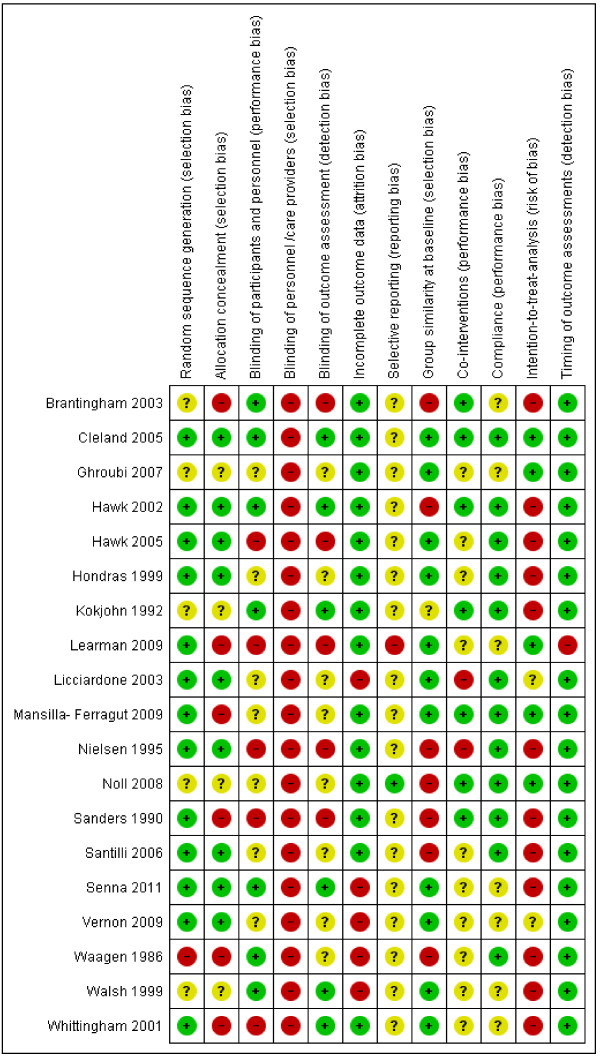
Risk of bias summary: review authors’ judgments about each risk of bias item for each included study.

Overall, high levels of agreement between review authors were achieved for risk of bias assessments with a Kappa of 0.84 (95% CI: 0.77 to 0.90) and a percentage of agreement of 89% (95% CI: 0.84 to 0.93). Kappa values ranged from 0.53 (for item 3 and 5) to 1.0 (for items 6, 7, and 12). The results of the RoB for the individual studies are summarized in Figure 2.

**Figure 3:**
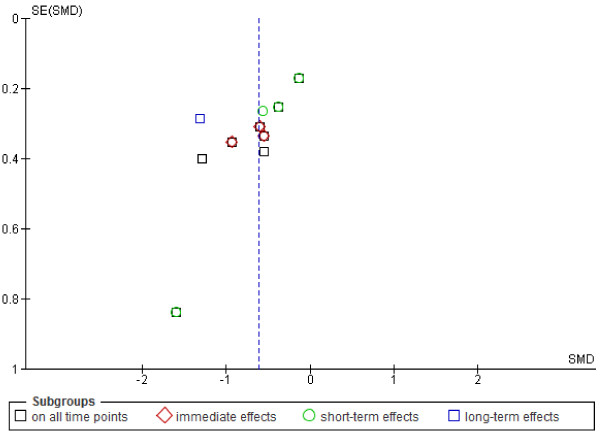
**Funnel plot of comparison: MT versus sham for the outcome pain.** Negative values favour MT.

Eight studies scored low risk of bias [23,25-28,31,33,36]. Due to the nature of the interventions it was not possible for care providers to be blinded. Intention- to- treat analysis scored negative or unknown in 14 studies (74%) [22,25-28,30,32,34-40]. Patients were successfully blinded in seven studies (37%) [22,23,25,28,36,38,39]. Selective outcome reporting, blinding of outcome assessors and co-interventions were the items most often judged as unclear. No firm conclusions could be drawn from the funnel plots that were suggestive of publication bias (Figure 3).

### Effect of manipulative therapy

The overall quality of the body of evidence is summarized in Table 2. We found moderate level of evidence for immediate effects of MT compared to sham for adults on pain. The subgroup analysis showed also moderate level of evidence for patients with musculoskeletal complaints on pain. All other levels of evidence were considered low to very low (Table 2).

**Table 2:** Summary of the overall quality of the body of evidence

**Type of outcome measure (population)**	**Time frame**	**(Number of studies) Number of participants**	**Summary of the quality of the evidence**	**Reasons for downgrading**
Pain (variety of complaints)	On all time points	(7RCTs) 389	Low evidence:	Risk of bias/imprecision
			MT provides better pain relief than sham	
	Immediate	(3RCTs) 117	Moderate evidence:	Incons
			MT provides better pain relief than sham	
	Short-term	(4RCTs) 272	Low evidence:	Risk of bias/imprecision
			MT provides better pain relief than sham	
	Long- term	(1RCT) 62	Low evidence:	Incons./imprecision
			MT provides better pain relief than sham	
Pain (musculoskeletal disorders)	On all time points	(5RCTs) 208	Low evidence:	Risk of bias/imprecision
			MT provides better pain relief than sham	
	Immediate	(2RCTs) 73	Moderate evidence:	imprecision
			MT provides better pain relief than sham	
	Short-term	(3RCTs) 135	Low evidence:	Risk of bias/imprecision
			MT provides better pain relief than sham	
	Long- term	(1RCT) 62	Low evidence:	Incons./imprecision
			MT provides better pain relief than	
Pain (neck pain)	Immediate	(2RCTs) 73	Low evidence:	Incons./imprecision
			MT does not provide better pain relief than sham	
Pain (low back pain)	Short-term	(2RCTs) 126	Low evidence:	Risk of bias/imprecision
			MT provides better pain relief than sham	
Pain (primary dysmenorrhea)	On all time points	(2RCTs) 181	Low evidence:	incons./imprecision
			MT does not provide better pain relief than sham	
Pain (chiropractor)	On all time points	(3RCTs) 190	Very low evidence:	Risk of bias/incons./imprecision
			MT does not provide better pain relief than sham	
Pain (physical therapist)	On all time points	(3RCTs) 137	Low evidence:	Risk of bias/imprecision
			MT performed by physical therapist provides better pain relief than sham	
Pain (physician)	On all time points	(1RCT) 62	Low evidence:	incons./imprecision
			MT performed by a physician therapist provides better pain relief than sham	
Disability (muscoloskeletal disorders)	Short term	(6RCTs) 355	Very low evidence:	Risk of bias/incons./imprecision
			MT does not provide better relief of disability than sham	
Perceived (asthma) recovery (chronic asthma)	Short term	(1RCTs) 31	Very low evidence:	Risk of bias/incons./imprecision
			MT does not provide better perceived (asthma) recovery than sham	

incons. = inconsistency.

#### ***Pain***

**Figure 4:**
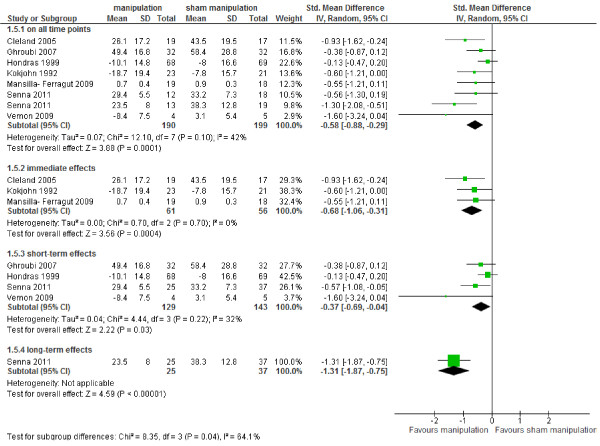
Forest plot of all studies comparing MT versus sham on pain.

Data of seven studies could be pooled [23,24,27,28,31,36,37], six studies did not provide data for calculating SMD or WMD [22,25,29,30,34,38]. Figure 4 shows that there is low level of evidence (high RoB, imprecision) that MT provided statistically significantly better pain relief than sham MT on all time points SMD -0.58 (95% CI – 0.88 to – 0.29) [23,24,28,31,36,37], and at short-term follow-up SMD - 0.37 (95% CI – 0.69 to – 0.04) [24,27,36,37]. We found moderate level of evidence (imprecision) that MT provided better pain relief than sham MT immediate after treatment SMD -0.68 (95% CI -1.06 to -0.32) [23,28,31]. There is low level of evidence from one study (inconsistency, imprecision) that MT is better than sham at long term follow-up SMD -1.31 (95% CI -1.87 to -0.75) [36]. The effects were considered clinically relevant on all time points, immediate after treatment and at long term follow-up.

#### ***Subgroup analyses***

The largest subgroup concerned patients with musculoskeletal disorders. In these patients there is moderate level of evidence (imprecision) that MT provided statistically significant and clinically relevant better pain relief than sham MT immediately after treatment SMD -0.73 (95% CI, -1.21 to -0.25) [23,31]. We found low level of evidence (high RoB, imprecision) for pain relief at short term follow-up SMD -0.52 (95% CI -0.87 to -0.17) [24,36,37], and low level of evidence (inconsistency, imprecision) at long term follow-up SMD -1.31 (95% CI -1.87 to -0.75) [36]. Moreover, there is low level of evidence (high RoB, imprecision) on all time points SMD -0.71 (95% CI -1.02 to -0.39) [23,24,31,36,37]. Two studies (213 participants) presented dichotomous data [26,35], and showed that MT provided better pain relief than sham: RD 0.27 (95% CI 0.11 to 0.42). These differences are considered clinically relevant.

Considering patients with low back pain, there is only low level of evidence (high RoB, imprecision) that MT showed statistically significantly better pain relief than sham MT (126 participants) at short term follow-up SMD -0.47 (95% CI -0.82 to -0.11) [24,36].

For neck pain patients, there is low level of evidence (inconsistency, imprecision) that MT provides better pain relief than sham immediately after treatment SMD -0.73 (95% CI -1.21 to -0.26) [23,31].

For non-musculoskeletal disorders, two low RoB studies (181 participants) with primary dysmenorrhea demonstrated a non-significant effect in favor of MT on pain relief WMD -5.31 (95% CI -13.62 to 2.99) [27,28]. There is low level of evidence that MT is no better than sham on pain relief in patients with dysmenorrhea.

**Figure 5:**
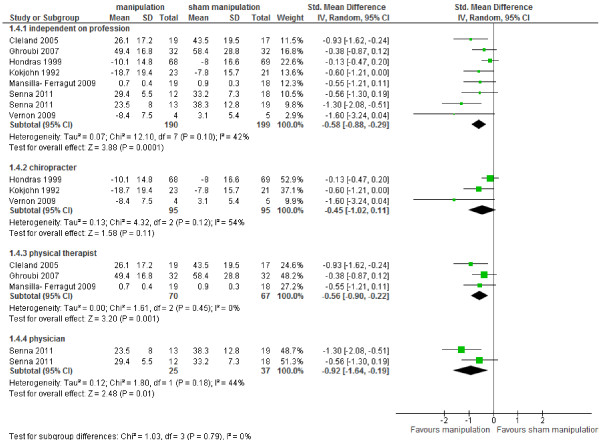
Forest plot comparing MT versus sham MT on pain stratified for profession.

Stratification for profession, yielded in no significant differences between the professions. MT performed by physicians provided somewhat lager effect sizes than the other professions (Figure 5), however, these results were based on one low RoB study [36].

#### ***Sensitivity analyses***

Sensitivity analyses did not change our main findings. Only at short term follow-up the level of evidence changed from low level of evidence for pain relief to moderate level of evidence for no significant differences between the groups. The pooled effect size (SMD) decreased from -0.37 (-0.69 to -0.04) to -0.30 (-0.72 to 0.11) [27,36].

For the subgroup musculoskeletal disorders, the level of evidence changed from low level of evidence for pain relief to moderate level of evidence for pain relief on all points. The SMD changed from 0.71 (-1.02 to -0.39) to -0.81 (95% CI -1.17 to -0.45) [23,31,36].

#### ***Disability***

Pooling was not possible because of statistical heterogeneity. There is very low level of evidence (high RoB, inconsistency, imprecision) that MT has no statistically significant effect on disability [22,24-26,30,36].

#### ***Perceived recovery***

One study with high risk of bias (31 patients with chronic asthma) evaluated perceived (asthma) recovery [32]. There is very low level of evidence (high RoB, inconsistency, imprecision) that MT has no statistically significant effect on perceived (asthma) recovery [32].

#### ***Quality of life***

Two studies (164 participants all with low back pain), one with low RoB, were included in the meta-analyses [35,36]. Data from two other studies could not be used [25,26]. There is very low level of evidence (high RoB, inconsistency, imprecision) that there is no statistically significant effect on quality of life MD 1.22 (95% CI, -7.24 to 9.67).

#### ***Range of motion***

Four studies (179 participants with musculoskeletal complaints), three with high RoB [22,38,40], evaluated range of motion (ROM) after MT [22,31,38,40]. Statistical pooling was not possible because of lack of data or heterogeneity on outcome. There is very low level of evidence (high RoB, inconsistency, imprecision) that MT is not more effective on ROM.

#### ***Pulmonary functions***

Pulmonary functions were evaluated in two studies (66 participants) [32,33]. Statistical pooling was not possible because lack on data [32]. There is low level of evidence (high RoB, imprecision) that MT does not provide better pulmonary functions.

#### ***Adverse events***

Eleven studies (58%) did not report about adverse events [22,24-26,28-30,34,38-40] while four studies reported no adverse events [23,31,32,35]. Adverse events in the MT group (9 participants) were limited to minor aggravation of neck pain or headache, muscle soreness, stiffness, tiredness, and local discomfort [27,33,36,37]. Also in the sham MT group some adverse events were reported (6 participants). These consisted of elevated blood pressure in the morning, mild heart palpitations and little muscle soreness [27,33]. None of the studies registered any serious complications in either the experimental or control group.

## Discussion

There is low to moderate level of evidence that MT has a significant effect on pain relief in adults with a variety of complaints and in the subgroup of patients with musculoskeletal disorders. Performing sensitivity analysis, including only studies with low Rob, did not change our main findings. Ideally we need interventions with immediate effects that preferably lead to long-term clinically relevant benefits. In this study we found benefit for MT, especially in patients with musculoskeletal disorders. The pooled effect estimates were considered clinically relevant.

A recent systematic review showed that musculoskeletal conditions were the most frequent indications for receiving spinal manipulation, with low back and neck pain being the most common ones [1]. Non-musculoskeletal conditions comprised a very small percentage of indications [1].

It appears reasonable that when MT is used there should be evidence for its efficacy with minimal or no harm. Only a few minor adverse events were reported in the included studies. There were no serious complications such as strokes. Sensitivity/subgroup analyses on the risk of specific manipulation techniques related to adverse events were not possible. Our findings are in agreement with earlier studies, which cast doubt about a causal relation between manipulation and stroke [11,12]. However, it must be acknowledged that the included trials were much too small to pick up more rare serious adverse events (if present).

Interestingly, this review found also some adverse events in the sham MT group [27,33]. Sham manipulation consisted of light touch at the same anatomic thoracic and occipital regions in the same position as the real manipulations [33], and low force maneuver at the left L2-L3 vertebral level in side lying position with bilateral flexion of the hips and knees [27]. Light touch is not expected to create physiological or biomechanical changes, therefore, we cannot explain these events. It seems that low force chiropractic techniques of at least 200 Newton may also produce some treatment effects and that these are indistinguishable from the real MT. To improve reporting of (minor) adverse events, we propose the usage of (validated) questionnaires, at all follow-up visits. An anonymous registration for practitioners in a database should be considered.

To our knowledge, there are no comparable systematic reviews that evaluated MT versus sham MT in adults with a variety of complaints. Therefore, we compared our results with systematic reviews, which evaluate MT on specific patient groups. An earlier systematic review on the effectiveness of MT for chronic low back pain patients found very low quality evidence that MT is equally effective than sham MT for short-term pain relief [15]. Their results were based on three RCTs [24,30,38], all included in this review. We added two more RCT, one with low RoB [29,36], resulting in a different conclusion: low evidence that MT showed statistically significantly better pain relief than sham MT. Our findings are in agreement with Gross et al 2010, who found low quality evidence for the use of thoracic manipulation for immediate pain relief in patients with neck pain [42]. A systematic review of spinal manipulations for patients with dysmenorrhea indicated that there was no evidence to suggest that spinal manipulation was effective in treating dysmenorrhea compared to sham, which is in line with our results [16]. Another Cochrane review for asthma reported from data of two trials [32,43] examining chiropractic MT compared to sham MT, that there are no significant differences between groups for lung function and quality of life measures [17]. One of the included trials concerned young (6 to 8 years) children and therefore was excluded from our systematic review [43].

Limitations of our review include the diversity of professions (chiropractor, physical therapist, osteopath or physician) who delivered the manipulations. Nevertheless, our subgroup analyses showed no clear differences in effect between different professions, but the power is low and the conclusion is based on 2 or 3 small studies. Another limitation is the diversity of sham manipulations. These varied from manipulations with a deactivated Activator instrument, a spring loaded piston activated instrument to low force mimic maneuvers or manual contact. A sham manipulation should produce the smallest possible treatment effect; because any manual intervention inevitably may produce some type of physiologic or biomechanical effect [44]. It is important that sham treatments are credible for the patient, equalizing the effect of expectation of improvement between groups, are valid, so that the patient can adequately be blinded. In this systematic review, adequate blinding of participants was performed in only seven studies [22,23,25,28,36,38,39]. Unclear and inadequate blinding may have affected and enlarged our pooled effect sizes. Moreover, blinding may be affected in patients previously exposed to manipulation.

Four studies used a cross-over design [29,32,39,40]. In crossover studies, participants will be aware eventually of the type of manipulations they received leading to probable bias and this may affect the outcome. Moreover, the effects of spinal manipulation cannot be reversed and are therefore likely to be carried over into the next cycle. However, these studies were not included in the meta-analyses and therefore, could not have affected our pooled results.

Most of our studies included less than 25 participants in their smallest study group. These studies could be considered as underpowered. Also, the overall power of the statistical pooling was limited. The total number of participants was less than 400 for continuous outcomes and 300 for dichotomous outcomes in all of our meta-analyses. Consequently, the level of evidence was downgraded. Our sensitivity analyses were comparable with the original analyses and showed that no other factors might have influenced the overall pooled effects.

Based on personal communication during the review process, two studies did not meet the inclusion criteria of manipulative therapy [25,26]. When asked, the original authors stated that no thrust was given. However, as we were unable to consequently contact all corresponding authors, we chose to base the study selection on the published reports and refrained from removing these studies from the manuscript. Nevertheless, excluding these studies [25,26] would not have affected our results as these were not included in the meta-analysis.

As in each systematic review, the possibility of publication bias cannot be omitted, and is more likely in small studies with non-significant results. Although, our funnel plots did not suggest that this was an issue in this review, relevant studies, hidden in unknown databases are difficult to locate and may not have been included. To reduce these biases, we performed a thorough search in multiple electronic databases and performed reference and hand-searching without language restrictions.

## Conclusion

### Implications for practice

MT produces pain relief immediate after treatment, at short- and long term follow-up, but no effects are found on disability and perceived (asthma) recovery. Clinicians could refer to MT for pain relief as a treatment goal. For patients with pulmonary diseases, no significant or clinical relevant effects were found.

### Implications for research

The quality of evidence varied from very low to moderate, indicating that further research is likely to have an impact on the confidence in the estimate of effect and is likely to change this estimate. There is a need for future low risk of bias RCTs with large sample sizes that evaluate the effect immediate after treatment and at short- and long term follow-up not only on pain but also on disability and perceived recovery. Moreover there is a need for evaluating the effect of these procedures on specific subgroups of patients with musculoskeletal disorders. Adverse events should be reported more consequently.

## Competing interests

The authors declare that they have no competing interests.

## Authors’ contributions

Contributors: GGMSP, KV, APV conceived and designed the study. GGMSP, ET determined eligibility of search results and scored risk of bias. GGMSP, ET, SK, MB extracted data from included studies. GGMSP, ET, APV analyzed and interpreted the data. GGMSP drafted the manuscript. BWK commented on a draft-version of the manuscript. All authors critically revised the manuscript and approved the final version to be published. This article was commissioned and externally peer reviewed. All authors read and approved the final manuscript.
